# Paediatric head and neck malignant neoplasms: A brazilian retrospective study

**DOI:** 10.4317/medoral.25614

**Published:** 2023-01-15

**Authors:** Wallena Albuquerque da Cunha, Ana Carolina Pedro Corazza, Karla Mayra Rezende, Marcelo Bönecker, Marina Gallottini

**Affiliations:** 1 Orcid: 0000-0002-3219-1273. PhD student. Department of Stomatology, School of Dentistry, University of São Paulo, SP, Brazil; 2 Orcid: 0000-0002-7711-3076. PhD student. Department of Paediatric Dentistry, School of Dentistry, University of São Paulo, SP, Brazil; 3 Orcid: 0000-0003-4340-0699. PhD student. Department of Paediatric Dentistry, School of Dentistry, University of São Paulo, SP, Brazil; 4 Orcid: 0000-0001-9786-6473. Full Professor. Department of Paediatric Dentistry, School of Dentistry, University of São Paulo, SP, Brazil; 5 Orcid: 0000-0001-6071-5110. Full Professor. Department of Stomatology, School of Dentistry, University of São Paulo, SP, Brazil

## Abstract

**Background:**

To assess the prevalence of oral and maxillofacial malignant neoplasias in children and adolescents diagnosed through biopsies sent to the Oral Pathology Laboratory at the University of Sao Paulo School of Dentistry.

**Material and Methods:**

A retrospective analysis of anatomopathological reports on patients between 1 and 18 years old issued by the oral and maxillofacial pathology laboratory between 1997 and 2021 was performed for demographic data, lesion site, type of biopsy, diagnostic hypothesis and final diagnosis.

**Results:**

The laboratory issued 76,194 anatomopathological reports during this period, of which 10.77% were of children and adolescents. Of this total, only 32 biopsies (32/8.204; 0.39%) were neoplasias in children and adolescents. Sarcomas were the most prevalent malignant neoplasms (19/32; 59%), followed by carcinomas (7/32; 22%), lymphomas (5/32; 16%) and ganglioneuroblastomas (1/32; 3%). Of these 32 patients, the most affected individuals were aged between 4 and 11 years old (47%), 18 (56%) were male, and the mandible was the main anatomical site involved (28%). In 41% of the cases (13/32), the diagnostic hypothesis of the biopsied lesion was mistakenly considered benign and there was no diagnostic hypothesis in 18% of the cases.

**Conclusions:**

Oral and maxillofacial malignant neoplasms in children and adolescents are uncommon and the accuracy of provisional diagnoses is low in these cases. Better knowledge on oral and maxillofacial malignant lesions in this population would help professionals to reduce the diagnostic time and consequently improve the patient’s prognosis.

** Key words:**Oral cancer, paediatric, adolescent, epidemiology, head and neck neoplasms, biopsy.

## Introduction

Cancer is a main cause of death by disease among children and adolescents aged 1 to 19 years old. In Brazil, according to the National Cancer Institute (INCA), it is estimated that there are 8,460 new cases of malignant neoplasms per year in this age group [1]. The most frequent types are leukaemia, central nervous system tumour, lymphomas and solid tumours, such as neuroblastoma and sarcomas (1-3).

Epidemiological studies aimed at assessing the prevalence of malignant neoplasms diagnosed with a biopsy of the oral and maxillofacial sites in children and adolescents have been performed in several continents. It is estimated that the occurrence of malignant neoplasms ranges from 0.5 to 2% in this age group (4-18). However, it is difficult to directly compare the results found because the study samples vary in terms of database size, age group, study period, tumour location and classification of the diseases into subgroups. Table 1 outlines some findings of the studies in the literature.

**Table 1:**
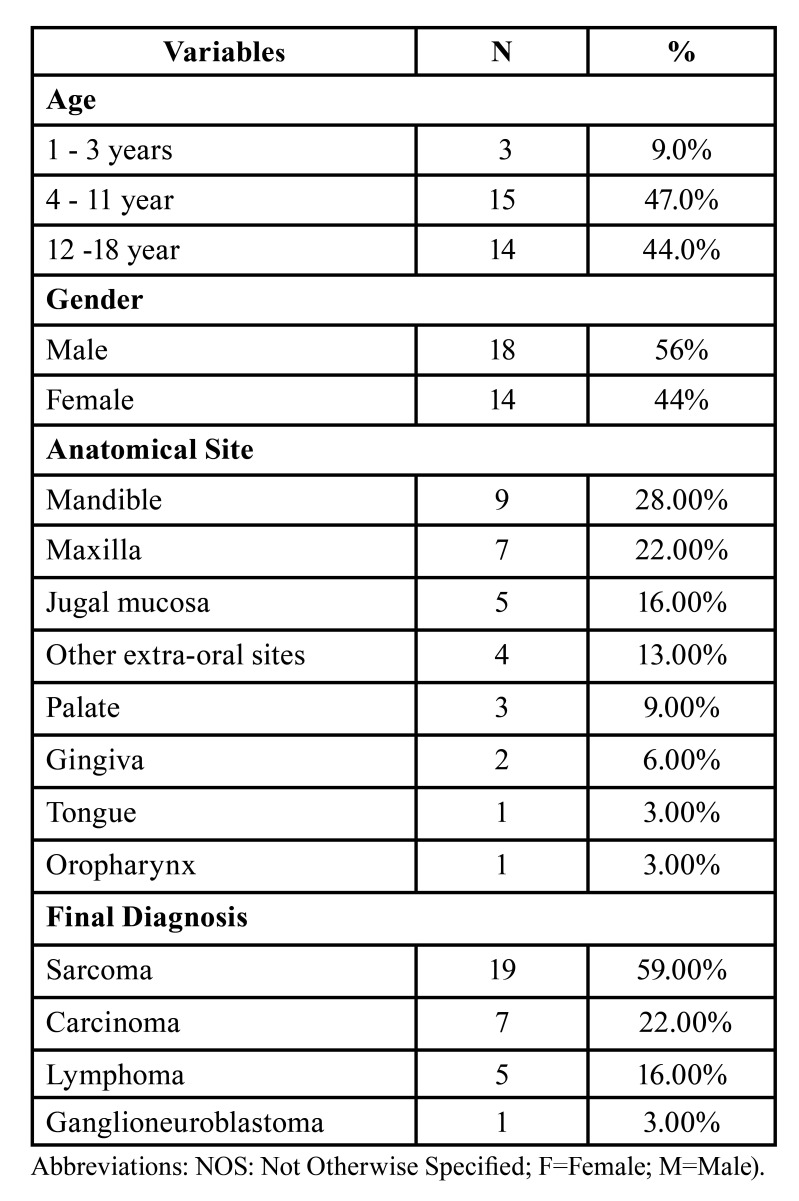
Malignant oral and maxillofacial lesions biopsied in children and adolescents from different geographic regions of the world.

It is necessary to reinforce the importance of epidemiological surveys on malignant neoplasms in the oral and maxillofacial sites to determine the prevalence of lesions and clinical-demographic characteristics of the affected population. Furthermore, knowledge of oral and maxillofacial neoplasms affecting children and adolescents can help paediatric dentists and general practitioners to identify these lesions, thus aiding in the diagnostic process. Therefore, this study aimed to assess the prevalence of oral and maxillofacial malignant neoplasms in children and adolescents diagnosed with biopsies.

## Material and Methods

The biopsy records and respective anatomopathological reports issued between 1997 and 2021 were obtained from the Oral Pathology Laboratory of the University of Sao Paulo School of Dentistry. In addition, demographic data on the patient were collected, including information on the lesion (i.e. site, evolution time), biopsy type (incisional or excisional), diagnostic hypotheses (benign or malignant lesion) and final diagnosis given by the pathologists.

The final diagnoses were established according to the 2017 World Health Organization classification of head and neck tumours which was used in the studies by Da Silva *et al*. (2019) (4), Silva *et al*. (2018) (9). De Carvalho *et al*. (2020) (10), and De Arruda *et al*. (2017) (13). The patients were divided into three age groups as follows: 0-3 years old (babies and toddlers), 4-11 years old (children) and 12-18 years old (adolescents). The anatomical sites involved were classified into maxilla, mandible, palate, jugal mucosa, lips, gingiva, tongue, oropharynx and others.

At the moment of this study, all the histological slides of malignant neoplasms were reviewed by two oral pathologists, except for one case (#7) of a diagnosis of ganglioneuroblastoma whose slide and paraffin block were not found in the records.The collected data were tabulated in electronic spreadsheet (Excel® Microsoft) for descriptive and quantitative analysis by using the Open Source Jamovi® software, version 1.6.23.

## Results

During the period between 1997 and 2021, the oral and maxillofacial pathology laboratory issued 76,194 histopathological diagnostic reports, of which 10.77% (8,204/76,194) were of children and adolescents. Of this total, 32 biopsies (32/8,204; 0.39%) received a final diagnosis of malignant neoplasms.

Of these 32 cases, 56% (n = 18) occurred in male patients and 44% (n = 14) in female ones. The highest frequency of neoplasms was observed in the age group of 4-11 years old (47%); *n* = 15), as shown in Table 2.

**Table 2:**
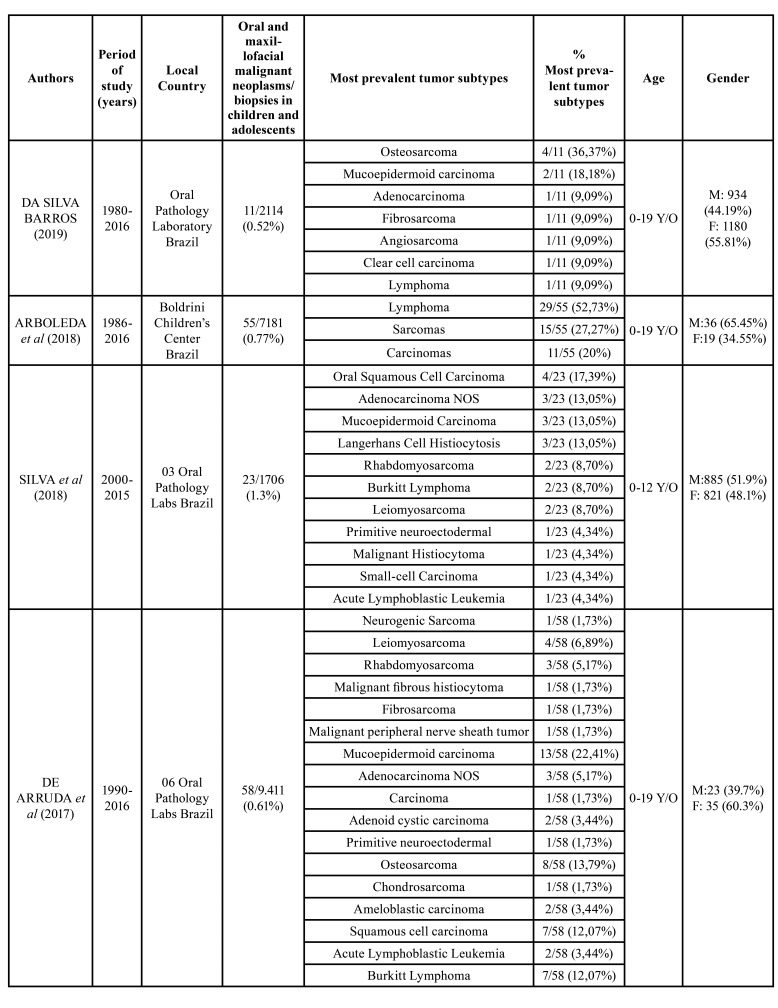
Demographic data and distribution of the oral and maxillofacial lesions.

**Table 2 cont.**
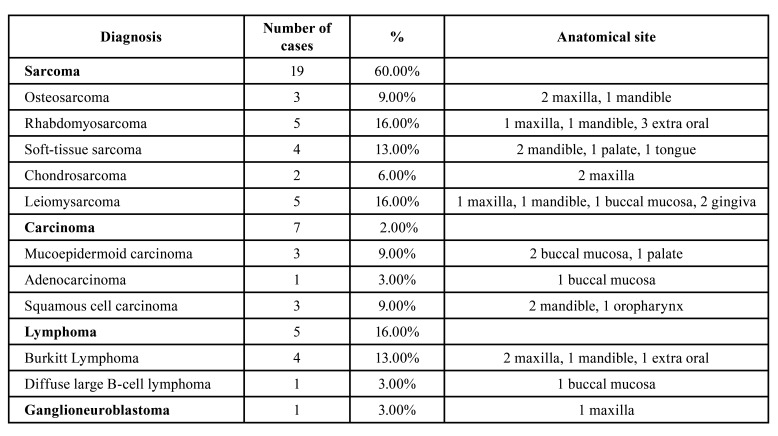
Demographic data and distribution of the oral and maxillofacial lesions.

**Figure 1:**
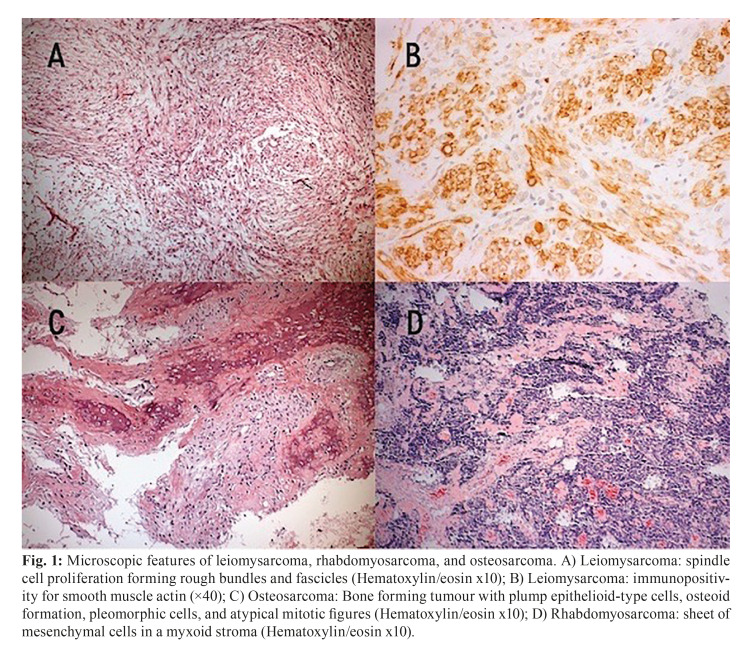
Microscopic features of leiomysarcoma, rhabdomyosarcoma, and osteosarcoma. A) Leiomysarcoma: spindle cell proliferation forming rough bundles and fascicles (Hematoxylin/eosin x10); B) Leiomysarcoma: immunopositivity for smooth muscle actin (×40); C) Osteosarcoma: Bone forming tumour with plump epithelioid-type cells, osteoid formation, pleomorphic cells, and atypical mitotic figures (Hematoxylin/eosin x10); D) Rhabdomyosarcoma: sheet of mesenchymal cells in a myxoid stroma (Hematoxylin/eosin x10).

The most prevalent malignant neoplasms were sarcomas (59%; *n*=19/32), especially leiomysarcoma (16%; *n*=5/32) and rhabdomyosarcoma (16%; *n*=5/32), followed by carcinomas (22%; *n*=7/32), lymphomas (16%; *n*=5/32) and ganglioneuroblastomas (3%, *n*=1/32). Among the seven cases of carcinomas, three were mucoepidermoid carcinoma (3/7), three were oral squamous cell carcinomas (3/7), and one was an adenocarcinoma (1/7) (Fig. 1).

The most affected anatomical site was the mandible (28%; 9/32), followed by maxilla (22%; 7/32), buccal mucosa (16%; 5/32), other sites (13%; 4/32), palate (9%; 3/32), gingiva (6.3%; 2/32), tongue (3%; 1/32) and oropharynx (3%; 1/32), as shown in Table 3.

The diagnostic hypotheses of the biopsied lesions raised by the dentist were benign in 41% of the cases (13/32) and malignant in 41% of the cases too (13/32), whereas there was no diagnostic hypothesis in 18% of the cases (6/32).

**Table 3:**
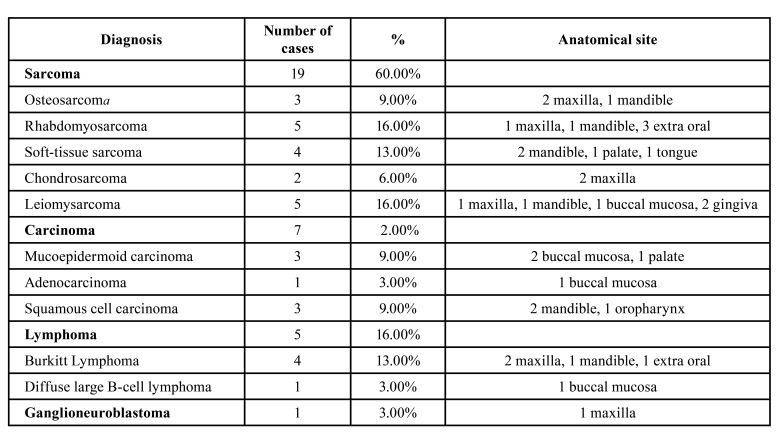
Diagnoses and anatomical sites.

## Discussion

The Oral Pathology Laboratory of the University of Sao Paulo School of Dentistry is the most outstanding oral pathology laboratory in Brazil and one of the largest in the world, receiving samples from the whole country. Since the beginning, it has already received more than 100 thousand samples of oral lesions. These findings certainly represent the Brazilian reality regarding the epidemiology of malignant neoplasms biopsied from the oral and maxillofacial sites of affected children and adolescents.

Our results showed that malignant neoplasms represented 0.39% (32/8,204) of the biopsies in children and adolescents which have been sent to our laboratory since 1997 and 10.77% (32/76,194) of all biopsies received, regardless of the patient’s age. This Figure of 0.39% is similar to that found by Ataíde *et al*.(14) (0.4%), Da Silva Barros(4) (0.52%) and rather smaller than that reported by De Arruda *et al*.(13) (0.61%), Jones & Franklin (5) (0.70%), Arboleda *et al*.(3) (0.77%), Dhanuthai, Banrai & Limpanaputtajak (1) (0.88%) and Wang *et al*.(8) (0.88%). However, we observed a large difference in a study conducted in a reference centre for paediatric oncology surgery in Southern Taiwan, 2.1% (6).

In the present study, the majority of malignant neoplasms were of mesenchymal origin (17/32), a finding also reported by previous studies (1,4,10,12,17).

Five cases of rhabdomyosarcoma were found. This malignant neoplasm of soft tissues is considered to be common in children, accounting for 40% of the cases involving the head and neck region. The development of drug and surgical treatments has currently been ensuring a better survival of the patients, but early diagnosis is necessary for a better prognosis (19-22).

In the present study, the mean age of the patients affected by rhabdomyosarcoma was 5 years old. Two cases were diagnosed in the early childhood (0-3 years), three in the childhood and only one in the adolescence (12-18 years). The literature corroborates this finding regarding age, which shows that the prevalence of rhabdomyosarcoma is higher in patients aged between 2 and 5 years old (23).

The sample of patients with rhabdomyosarcoma was homogeneous regarding gender, a finding in disagreement with the literature as studies report a predilection for male individuals (23). Also, the literature shows that this malignant neoplasm occurs more in the head and neck region, involving anatomical sites like orbits, parameninges and mouth. In this sense, our sample was rather discrepant as two cases were in the oral region (one in the maxillary region and the other close to the nasolabial region), one in the paranasal region and one in the infra-orbital region (19,23-25).

The diagnosis of all cases of rhabdomyosarcoma was confirmed through immunohistochemistry. The neoplastic cells of rhabdomyosarcoma were positive for vimentin, desmin, myoglobin and muscle-specific actin.

Among the 8,204 biopsies, only five were leiomysarcoma. It is a rare mesenchymal malignant neoplasm (soft tissue sarcoma) rarely affecting head and neck as well as the oral cavity (3%-10%). The etiopathogenesis of leiomysarcoma is uncertain due to its aggressive growth and dissemination pattern, but some studies have pointed to an association with trauma, hormone stimulation, previous cancer treatment with ionizing radiation, some virus (e.g. Epstein-Barr virus infections) and metastasize of a leiomysarcoma from other primary region (26,27).

The diagnosis is established by an incisional biopsy, followed by lesion excision with extended margin resection and anatomopathological analyses, including immunohistochemistry. The surgery can be combined with adjuvant radiotherapy or/and chemotherapy, and follow-up appointments (26,27).

In general, the immunohistochemical profile of the leiomysarcoma is positive for smooth-muscle-actin and muscle-specific antigen (HHF-35), showing a low-degree expression for desmin, but being negative for S-100 protein.

Our study reported three cases of Burkitt lymphoma in child patients (4-11 years old) and two cases in adolescent patients (12-18 years old), with mean age being similar to that described in the literature and a higher prevalence in male gender also corroborated elsewhere 28,29).

Non-Hodgkin lymphomas are considered to be more common in the head and neck region of paediatric populations, with Burkitt lymphoma being more prevalent in this group of patients and resulting in facial asymmetry, pain and mobility of teeth surrounding the lesion. Moreover, we can observe systemic manifestations such as abrupt loss of weight, headaches and fever. Burkitt lymphoma can be classified into three different forms as follows: endemic, when it is common in a paediatric population (ex: African children); sporadic, in which abdominal disease predominates, usually occurring in the region of ileocecal or mesenteric valve; and lastly, immunodeficiency-related, when it occurs in patients with acquired immunodeficiency and infected with Epstein-Barr virus (28,29).

Oral and maxillofacial carcinomas in paediatric patients are less frequently diagnosed when compared to adults. However, the present study found a prevalence of 22% (7/32) for these tumours. Mucoepidermoid carcinoma (3/7) and epidermoid carcinoma (3/7) were the most common types of carcinoma followed by adenocarcinoma (1/7). Due to the great expertise of the pathologists of our institution, part of our samples of carcinomas (3/7) came from other educational institutions for review and final diagnosis.

There were three cases of epidermoid carcinoma in the oropharynx, in which two were in 17-year-old adolescents and one in an 11-year-old child, probably related to HPV.

Age stratification was made to facilitate epidemiological analysis in the present study, in which children and adolescents are those individuals younger than 18 years old. The majority of biopsies (47%; 15/32) were performed in patients between 4 and 11 years old and their final diagnoses included sarcoma, carcinoma and lymphoma. Only three cases were observed before the age of 3 years, probably because invasive procedures are usually avoided in the early childhood when suspected benign lesions can be monitored and biopsied in a later time.

The present study has shown the disease’s predilection for male gender (56%). In this sense, however, there have been different findings as some studies report a similar prevalence between genders (7,17), whereas others report male prevalence (6) and female prevalence (18).

Our results show that in 41% (13/32) of the cases there was no concordance between the diagnostic hypotheses raised by the clinician and the final diagnosis issued by the pathologist regarding the malignancy of the lesion to be biopsied. In addition, in six cases (6/32; 18%) the clinicians recorded no diagnostic hypothesis on the biopsy form. This finding is of concern as the diagnostic hypothesis raised by the clinician who performs the biopsy will usually guide the final histopathological diagnosis, in addition to determining the correct type of biopsy to be performed.

Incisional biopsy is indicated even for small lesions when the hypothesis of malignancy is considered. However, performing an excisional biopsy to remove a malignant lesion may pose a problem as a further intervention will be required to at least widen the tumour-free margins. This, in many cases, can make it challenging to carry out a salvage surgery for the effective treatment of malignant neoplasms.

Therefore, knowing the prevalence of malignant neoplasms in children and adolescents allows the dentist to diagnose oral and maxillofacial lesions in this population assertively [4,10,18].

## Conclusions

We have concluded that oral and maxillofacial malignant neoplasms in children are rare and that sarcomas are more prevalent. Furthermore, the diagnostic hypothesis on the nature of the lesion was correct in only 41% of the cases, which indicates that dentists are lacking preparation for this theme.
